# Supplementary material for: Genome-Wide Characterization and Expression Profiling of GASA Genes during Different Stages of Seed Development in Grapevine (Vitis vinifera L.) Predict Their Involvement in Seed Development
Source: Int J Mol Sci. 2020 Feb 6;21(3):1088. doi: 10.3390/ijms21031088 (PMC7036793; doi:10.3390/ijms21031088)
Supplement: Supplementary file 1 [file ijms-21-01088-s001.pdf]

**Supplementary Figure 1**

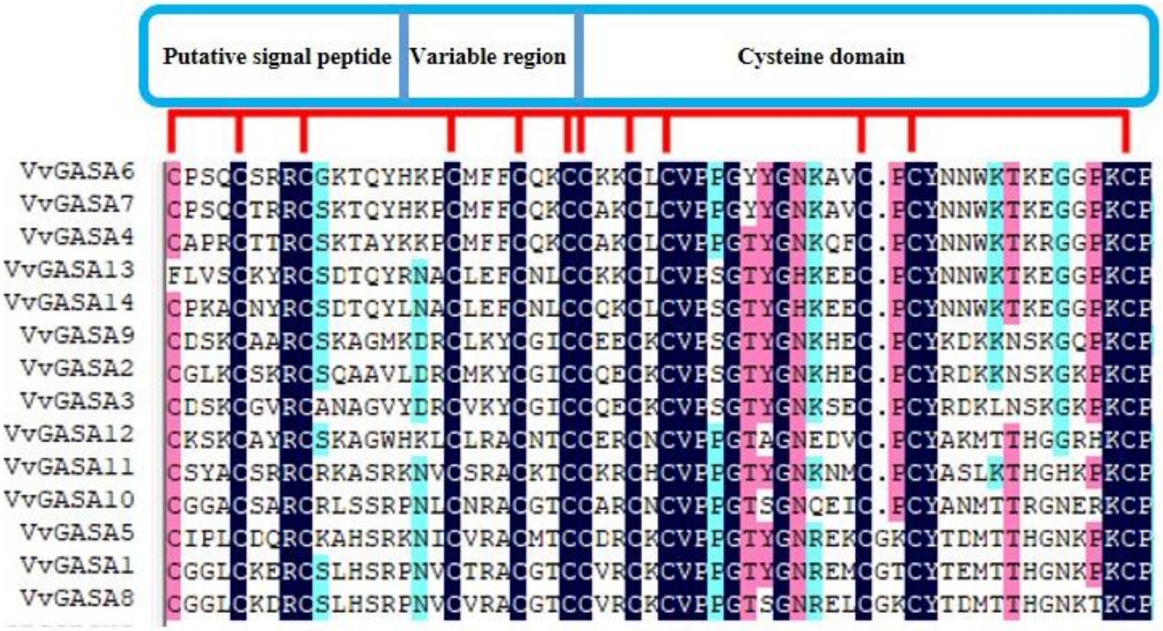


**Table S1: Tandem duplication events in grapes GASAgenes**

| Cluster number | Chromosome | Gene | Start Site | End Site |
|---|---|---|---|---|
| 1 | 18 | VvGASA11 | 20718815 | 20720279 |
| | 18 | VvGASA12 | 20720304 | 20720676 |
| 2 | Un- | VvGASA13 | 9775242 | 9775609 |
| | Un- | VvGASA14 | 9791751 | 9792551 |

**Table S2:** Synteny blocks of GASA genes within grape genome

| Region 1 (Grape) | | | | Region 2 (Grape) | | | Gene in the synteny region | |
|---|---|---|---|---|---|---|---|---|
| ID① | Chr | Start | Stop | Chr | Start | Stop | Gene 1 | Gene 2 |
| 53 | 17 | 5814755 | 7662326 | 14 | 28457051 | 30137019 | VvGASA7 | VvGASA6 |
| 56 | 17 | 8423634 | 11518764 | 14 | 26536960 | 28478988 | VvGASA8 | VvGASA5 |
| 62 | 18 | 6826940 | 8000941 | 3 | 6548597 | 8827504 | VvGASA9 | VvGASA2 |
| 113 | 7 | 15058884 | 16256503 | 18 | 7338029 | 8296810 | VvGASA3 | VvGASA9 |

**Table S3**: Primer sequences used in expression analysis of GASA genes in grape

| Gene | Forward and reverse primer sequence (5'~3') | |
|---|---|---|
| VvGASA1 | F | GGCTTCTGCCTTTCTTGGA |
| | R | GGTGGTCATCTCGGTGTAG |
| VvGASA2 | F | CTCCCTTCTCCTTCACTCC |
| | R | GCTTGTTCCCATACGTCC |
| VvGASA3 | F | GGTCCACCCACCACAGC |
| | R | GCACCTCACCCCACACTT |
| VvGASA4 | F | CTCCACAGCCCCAACAA |
| | R | AGGCACGCACAAGCACT |
| VvGASA5 | F | CCAGCAATGAGGAGTAGGTT |
| | R | GGTGTCTTGTCTTTTTGGATAGT |
| VvGASA6 | F | TCTCTTGGCTCTTCTCGC |
| | R | GGAACGCACAGGCACTT |
| VvGASA7 | F | GAATCCAAATCAAGCCCAC |
| | R | GGACCATACGACCCACTG |
| VvGASA8 | F | CAAGACATAGATTGCGGAGG |
| | R | CGACCCAGTTGGAAGAGAC |
| VvGASA9 | F | TCTCCCACCTGCCTTATTC |
| | R | GAGGGGCTTCATCTTTCT |
| VvGASA10 | F | AATGGATTGCGGGGGAG |
| | R | TCATGTTGGCGTAGCAGG |
| VvGASA11 | F | CCTCTTCTTCACCCACTTTC |
| | R | CATCTCTGCTTTCATCCACACT |
| VvGASA13 | F | TTATTTCCTTGTGTCATGCA |
| | R | ACCCTCCTTGGTCTTCC |
| VvActin | F | GATTCTGGTGATGGTGTGAGT |
| | R | GACAATTTCCCGTTCAGCAGT |
